# Supplementary material for: Single-cell transcriptomic atlas of the human retina identifies cell types associated with age-related macular degeneration
Source: Nat Commun. 2019 Oct 25;10:4902. doi: 10.1038/s41467-019-12780-8 (PMC6814749; doi:10.1038/s41467-019-12780-8)
Supplement: Supplementary file 1 — Supplementary information [file 41467_2019_12780_MOESM1_ESM.pdf]

Supplementary Information

Supplementary Figures:

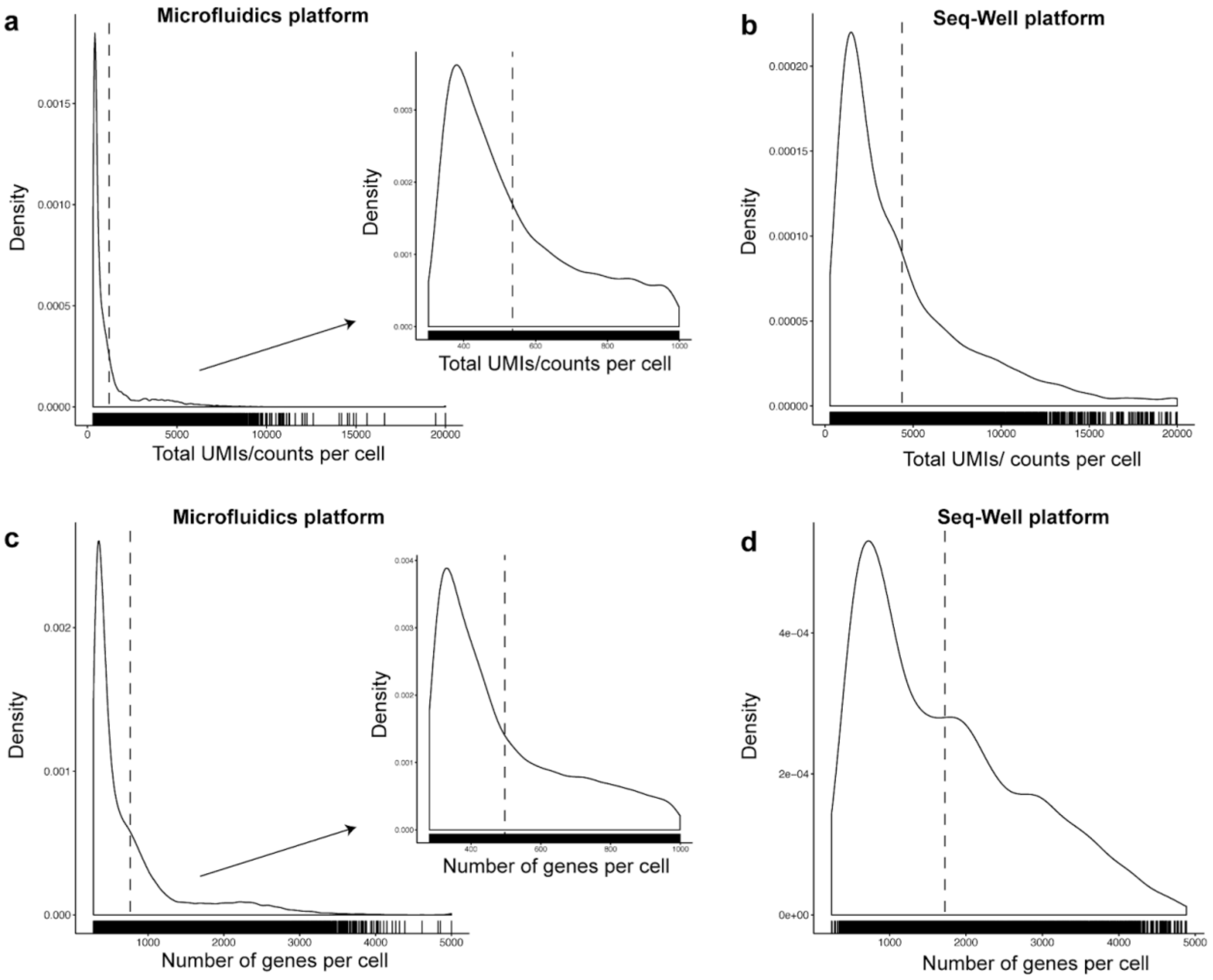

Supplementary Figure 1. Cell statistics post-processing. (a-b) Distribution of the total UMIs/counts per cell, with a mean of (a) 1,211 UMIs/counts per cell for microfluidics platform and (b) 4,834 UMIs/counts per cell for the Seq-Well platform. **(c-d)** Distribution of the number of genes identified with non-zero counts profiled per cell, with a mean of (c) 766 genes per cell for microfluidics platform and (d) 1,732 genes per cell for the Seq-Well platform.

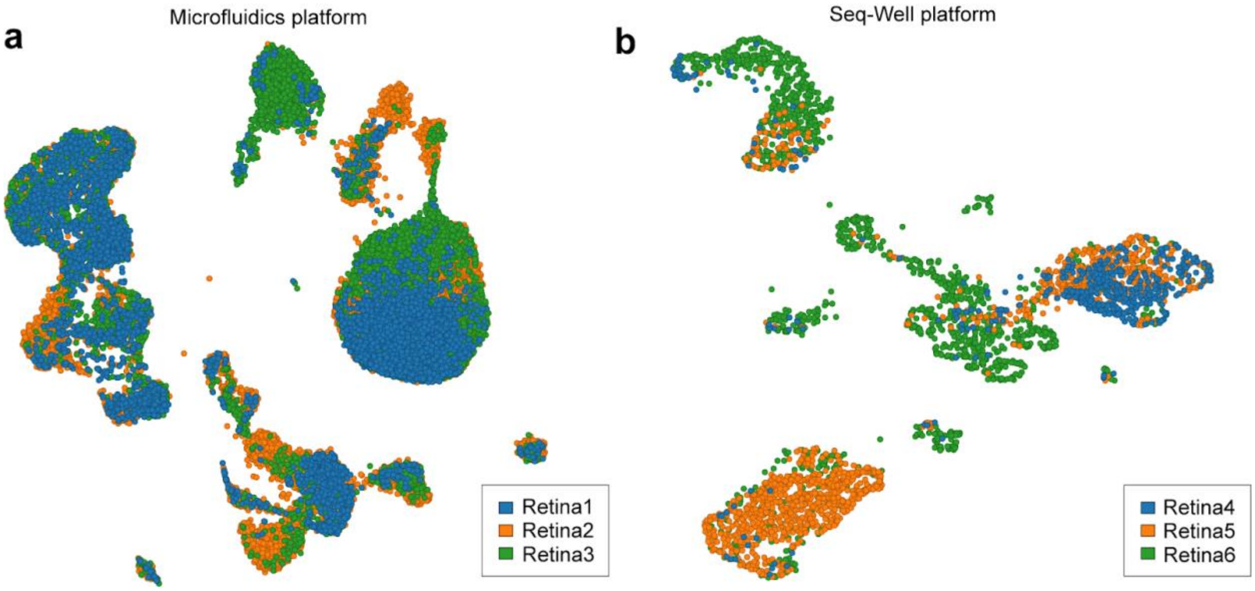

Supplementary Figure 2. Cellular distribution based on individual samples. Cell network plots showing the distribution of cells from the human retinal donors on either **(a)** microfluidics or **(b)** Seq-Well platform across all clusters.

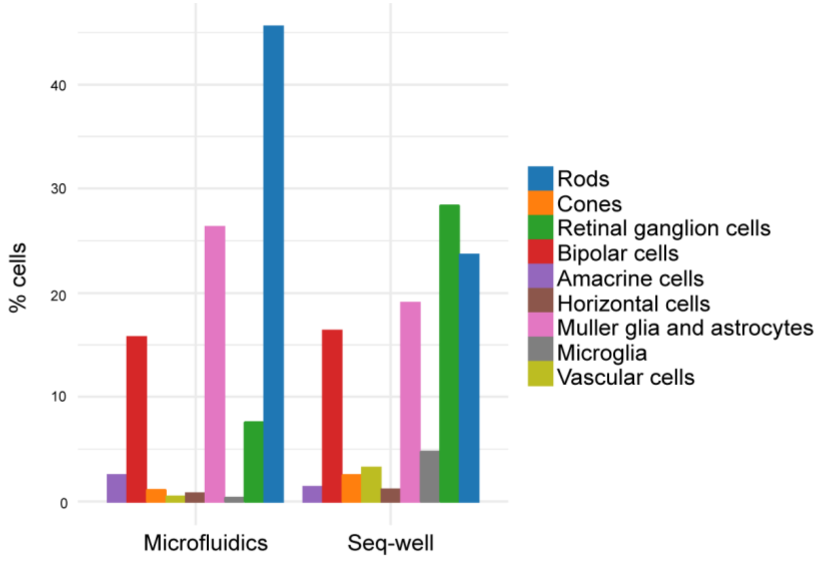

Supplementary Figure 3. Frequency of human retinal cell types identified by sequencing platforms. Bar graph showing the percentages of retinal cell types identified using the microfluidics (left) and Seq-Well (right) platforms.

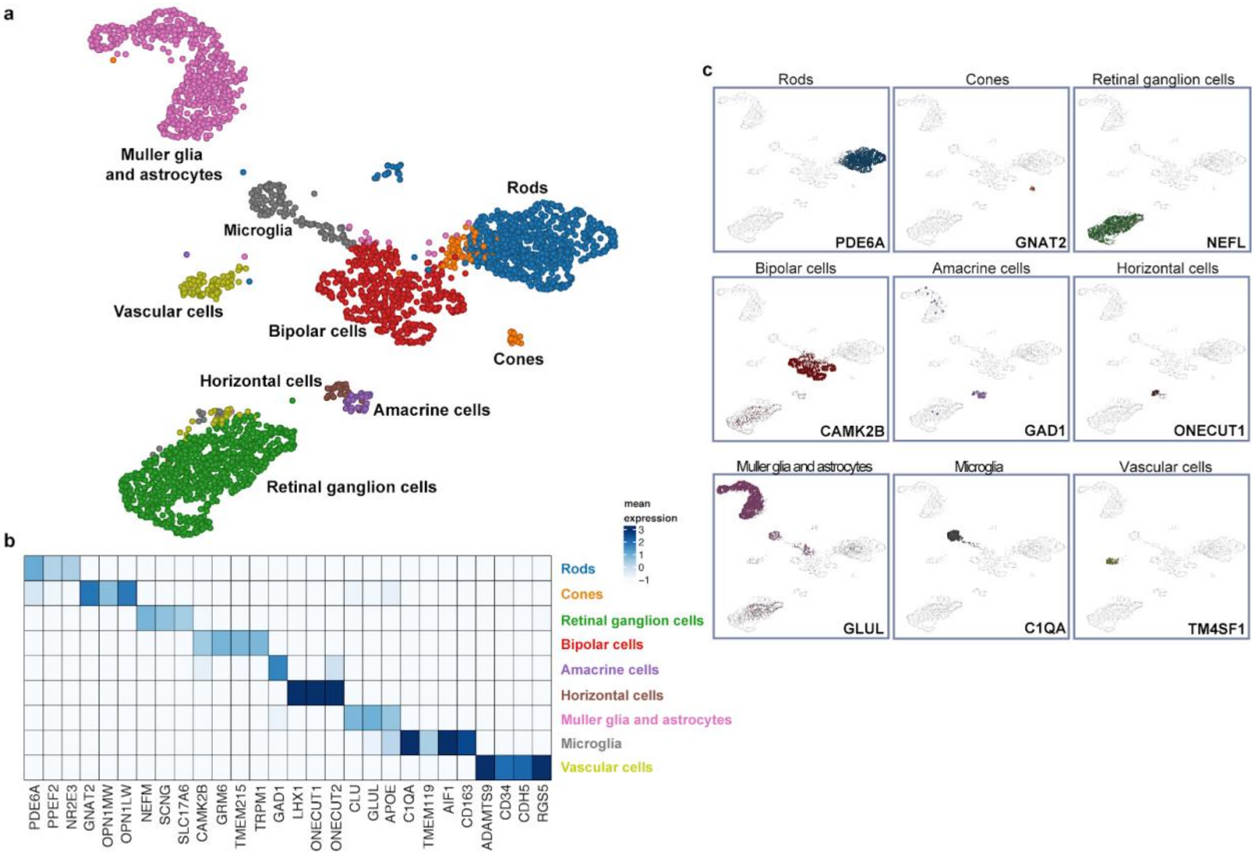

Supplementary Figure 4. Single-cell transcriptomic analysis of human retina using the Seq-Well platform. (a) Cell-to-cell similarity network layout of retinal cells sequenced using the seq-well platform. **(b)** Average expression of known cell-type marker genes across cell groups. **(c)** Projection of known cell-type-specific marker gene expression across cell groups. (b) and (c) show localization of distinct cell types within the network, identifying neighborhoods of rods, cones, retinal ganglion cells, bipolar cells, amacrine cells, horizontal cells, macroglia (Müller glia and astrocytes), microglia and vascular cells.

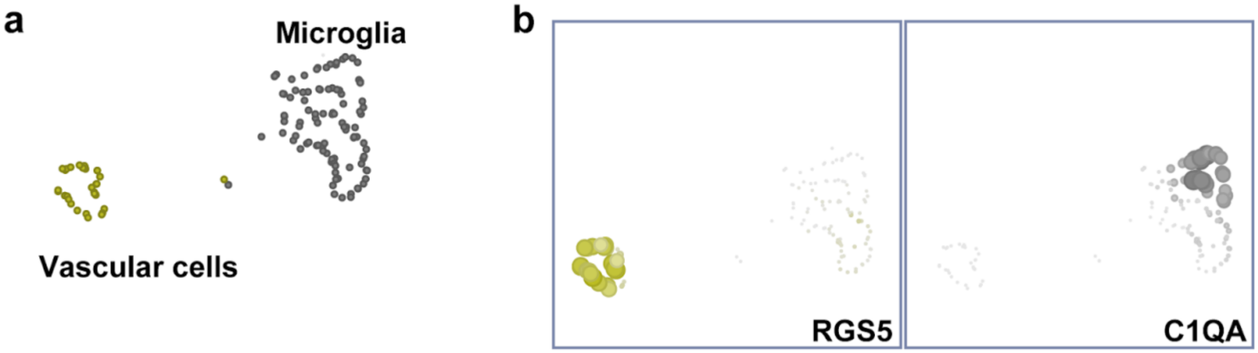

Supplementary Figure 5. SubACTIONet of vascular cells in the human retinal atlas. (a) Cells annotated as either vascular cells or microglia are extracted and a separate ACTIONet is constructed. Cells are then reannotated within the new subACTIONet. **(b)** Projection of known marker genes verifies the annotation of cells.

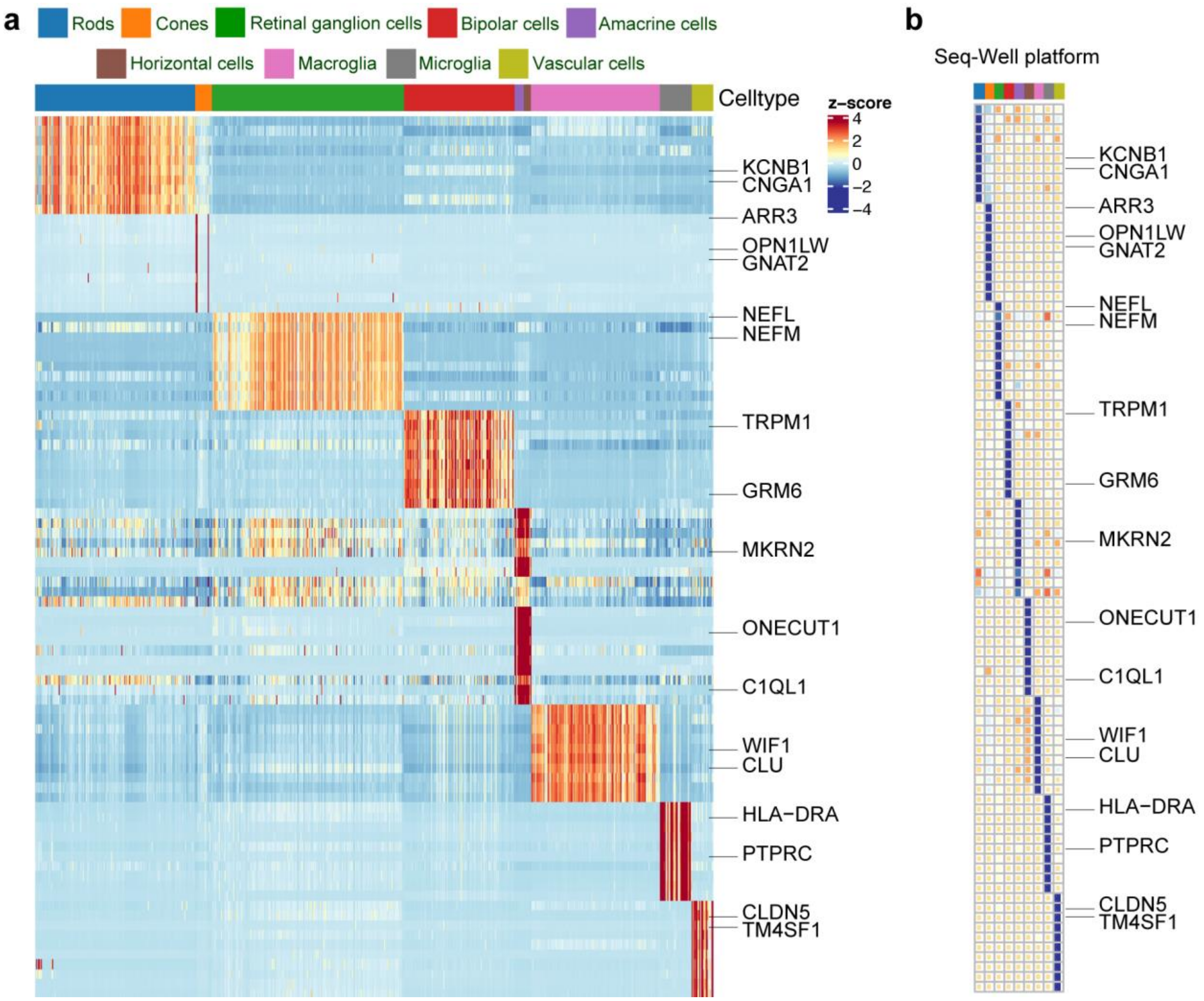

Supplementary Figure 6. Heatmap of the top marker genes within human retinal cell types (Seq-Well). The top differentially expressed genes were selected using the Wilcoxon rank-sum test and ranked based on their discriminating scores within each identified cell type. **(a)** imputed gene expression, using network diffusion, across all cells, **(b)** Average of raw expression of each gene within each cell type. In both panels, rows correspond to genes cell-types are color-coded (color bar, top) and marker genes (rows).

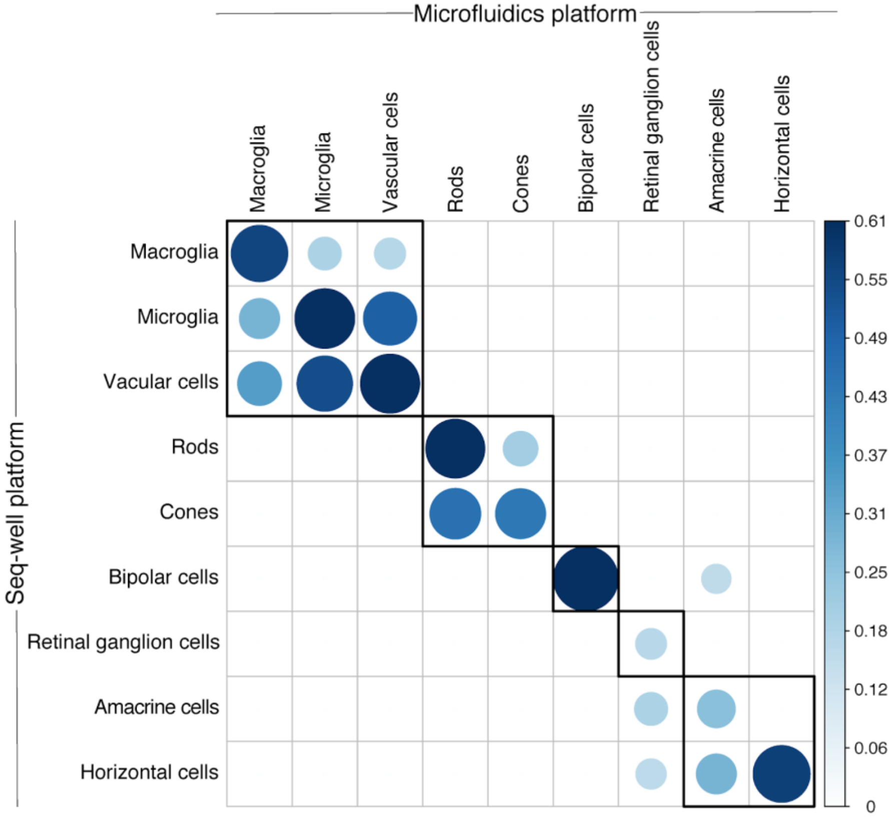

Supplementary Figure 7. Correlation between droplet-based microfluidics and Seq-Well platform. For each cell type in both platforms, a representative archetype has been selected (either from the main ACTIONet or corresponding subACTIONet) as its signature. Concordance of each pair of signatures is computed using Pearson's correlation and thresholded at 0.1 for visualization.

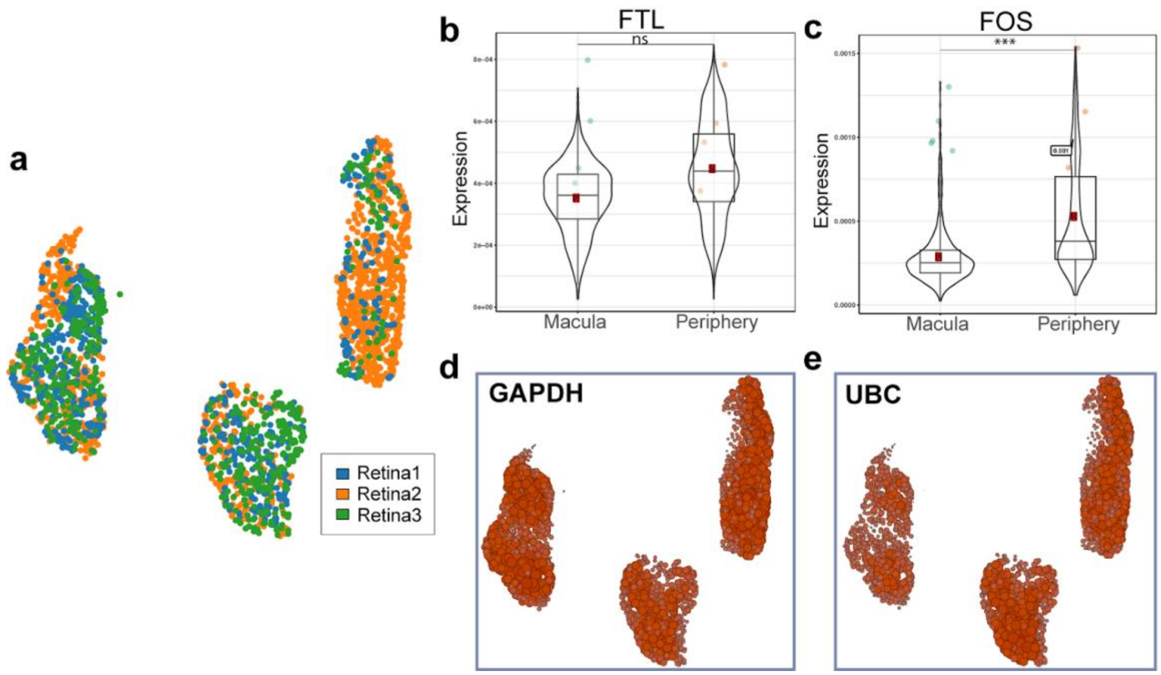

Supplementary Figure 8. Cellular distribution of Müller glia and astrocytes based on batches. (a) Cell network plots of microfluidics data showing the distribution of cells from the three human donors each as a function of postmortem interval on microfluidics platform. **(b-c)** The regional distribution of (b) FTL-subset and (c) FOS-subset of macroglial cells from the three human donors in the macula or peripheral retina. ns, not significant; ***p<0.001 **(d-e)** Expression of housekeeping genes (d) GAPDH and (e) UBC on the cell network.

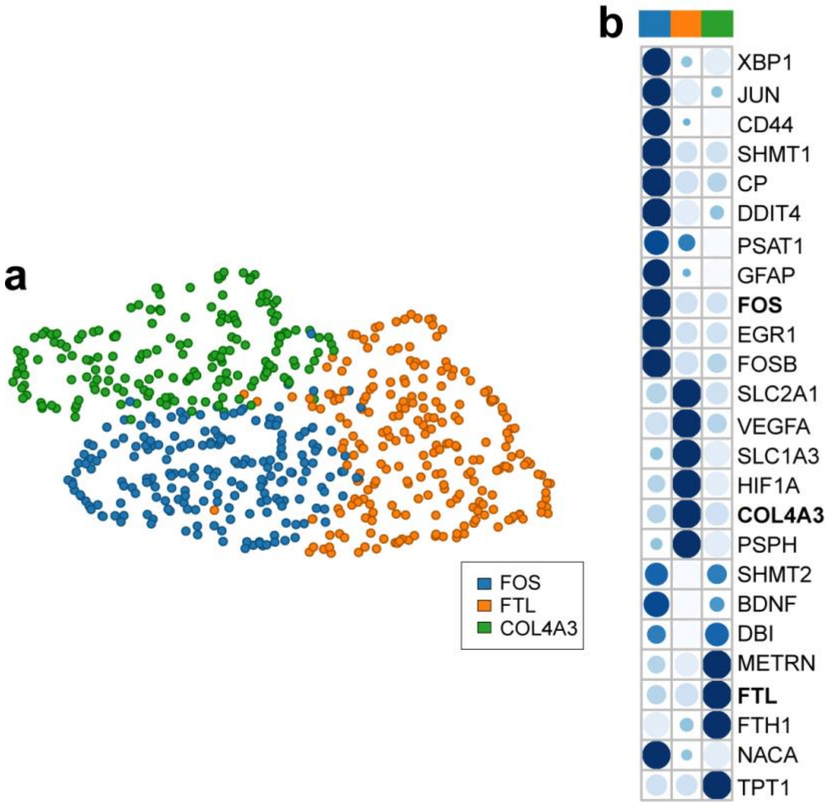

Supplementary Figure 9. Heterogeneity within Müller glia and astrocytes using Seq-Well platform. (a) Cell-to-cell similarity network (ACTIONet) representation of the filtered set of Müller glia and astrocytes, highlighting three major subtypes. **(b)** Dot plot showing the row-normalized (*z*-score) mean expression of the top-ranked genes across subtypes of macroglial cells.

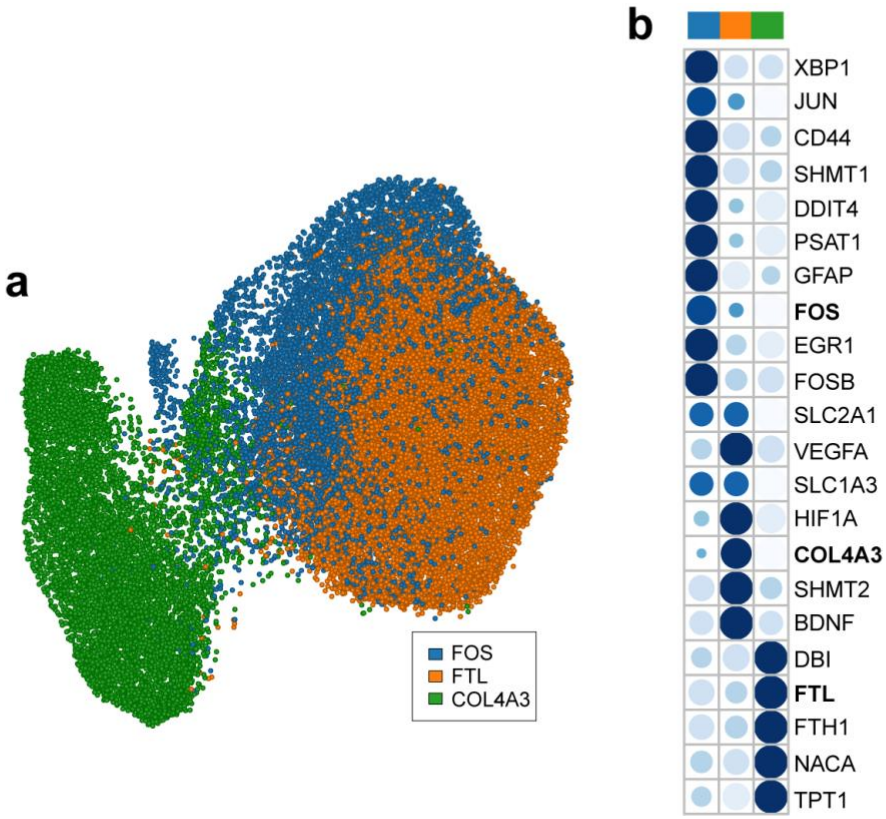

Supplementary Figure 10. Heterogeneity within non-human primate glia using macaque scRNA-seq dataset from Peng et al.[6] (a) Cell-to-cell similarity network (ACTIONet) representation of the filtered set of macroglia, highlighting three major subtypes. **(b)** Dot plot showing the row-normalized (*z*-score) mean expression of the top-ranked genes across subtypes of macroglial cells.

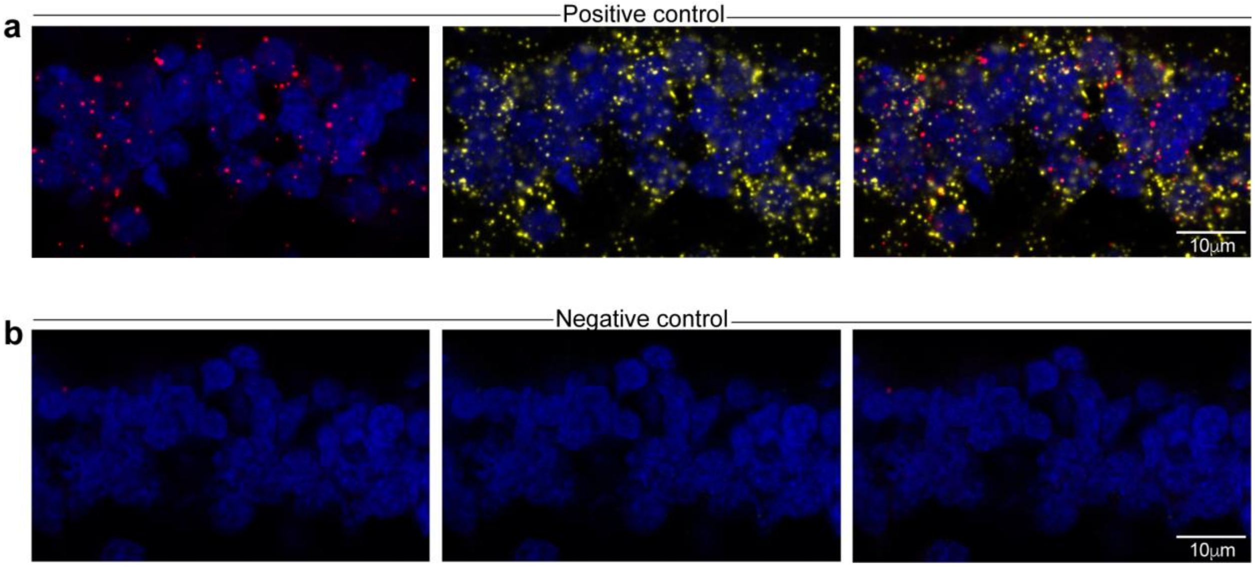

Supplementary Figure 11. Positive and negative control probes for fluorescence *in situ* hybridization. Representative images of multiplex *in situ* hybridization of **(a)** positive control probes (*POL2RA* labelled in red and *UBC* labelled in yellow) and **(b)** negative control probe (*DapB* labelled in yellow and red). Data is representative of three independent experiments.

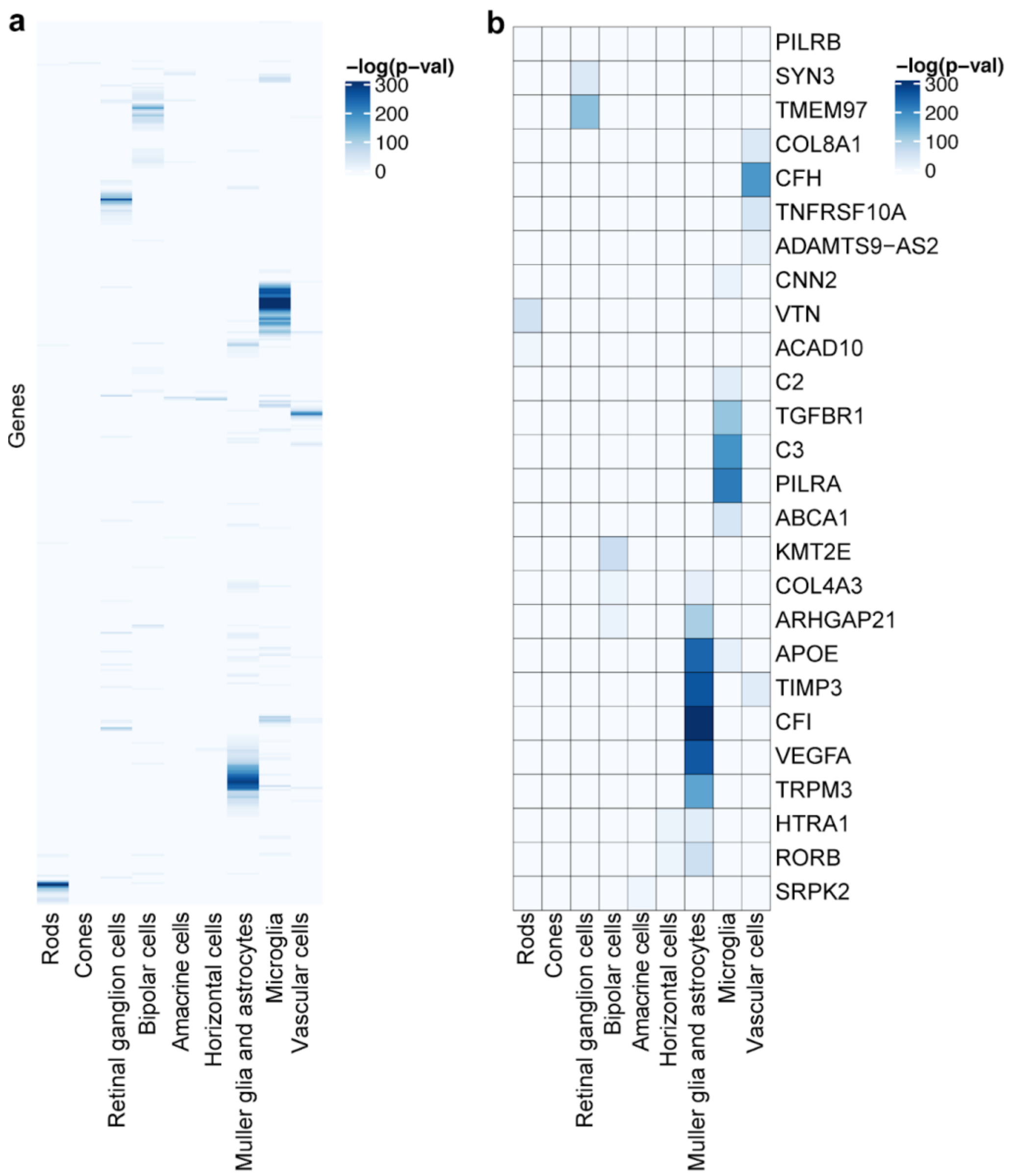

Supplementary Figure 12. Cell-type specificity of AMD-associated genes. Wilcoxon rank-sum test has been used to assess specificity of genes from the Seq-Well platform, within the full and lead subsets of AMD-associated genes, independently, across cell types. Reported specificity scores are the log-transformed value of adjusted *p*-values.

Supplementary Tables:

Supplementary Table 1. Human retinal specimen details.

Retina	Sex	Age	Postmortem Interval	Left/Right Eye	Assay
1	M	68	3.0	Right	Microfluidics scRNA-seq
2	M	71	3.2	Right	Microfluidics scRNA-seq
3	M	86	3.5	Left	Microfluidics scRNA-seq
4	M	65	24	Right	Seq-well scRNA-seq
5	F	66	8.0	Right	Seq-well scRNA-seq
6	M	86	6.0	Left	Seq-well scRNA-seq
7	M	74	17.0	Left	FISH, IF
8	M	78	4.3	Left	FISH, IF
9	M	84	6.0	Left	FISH, IF
10	M	85	3.4	Left	FISH, IF

scRNA-seq, single-cell RNA sequencing; FISH, fluorescence in situ hybridization; IF, immunofluorescence
