# Supplementary material for: Single-cell transcriptomic atlas of the human retina identifies cell types associated with age-related macular degeneration
Source: Nat Commun. 2019 Oct 25;10:4902. doi: 10.1038/s41467-019-12780-8 (PMC6814749; doi:10.1038/s41467-019-12780-8)
Supplement: Supplementary file 2 — Reporting Summary [file 41467_2019_12780_MOESM2_ESM.pdf]

nature research

Corresponding author(s): Brian P. Hafler

Last updated by author(s): Sep 23, 2019

# Reporting Summary

Nature Research wishes to improve the reproducibility of the work that we publish. This form provides structure for consistency and transparency in reporting. For further information on Nature Research policies, see Authors & Referees and the Editorial Policy Checklist.

## Statistics

For all statistical analyses, confirm that the following items are present in the figure legend, table legend, main text, or Methods section.

| n/a | Confirmed | |
|---|---|---|
| ☐ | ☒ | The exact sample size (*n*) for each experimental group/condition, given as a discrete number and unit of measurement |
| ☐ | ☒ | A statement on whether measurements were taken from distinct samples or whether the same sample was measured repeatedly |
| ☐ | ☒ | The statistical test(s) used AND whether they are one- or two-sided<br>*Only common tests should be described solely by name; describe more complex techniques in the Methods section.* |
| ☐ | ☒ | A description of all covariates tested |
| ☐ | ☒ | A description of any assumptions or corrections, such as tests of normality and adjustment for multiple comparisons |
| ☐ | ☒ | A full description of the statistical parameters including central tendency (e.g. means) or other basic estimates (e.g. regression coefficient) AND variation (e.g. standard deviation) or associated estimates of uncertainty (e.g. confidence intervals) |
| ☐ | ☒ | For null hypothesis testing, the test statistic (e.g. *F*, *t*, *r*) with confidence intervals, effect sizes, degrees of freedom and *P* value noted<br>*Give P values as exact values whenever suitable.* |
| ☒ | ☐ | For Bayesian analysis, information on the choice of priors and Markov chain Monte Carlo settings |
| ☒ | ☐ | For hierarchical and complex designs, identification of the appropriate level for tests and full reporting of outcomes |
| ☐ | ☒ | Estimates of effect sizes (e.g. Cohen's *d*, Pearson's *r*), indicating how they were calculated |

*Our web collection on statistics for biologists contains articles on many of the points above.*

## Software and code

Policy information about availability of computer code

| | |
|---|---|
| Data collection | Single cell RNA sequencing data was generated using the Illumina NextSeq 500 according to the manufacture's instructions. |
| Data analysis | To analyze data, we used publicly available software, Cell Ranger Software (version 3.0.2; 10X Genomics), bcl2fastq2 (version 2.19.1), STAR aligner, R, Multi-marker Analysis of GenoMic Annotation (MAGMA), and ImageJ. Custom code used in bioinformatics analyses is available at http://compbio.mit.edu/ACTIONet/, https://github.com/shmohammadi86/ACTIONet, and upon request. |

For manuscripts utilizing custom algorithms or software that are central to the research but not yet described in published literature, software must be made available to editors/reviewers. We strongly encourage code deposition in a community repository (e.g. GitHub). See the Nature Research guidelines for submitting code & software for further information.

## Data

Policy information about availability of data

All manuscripts must include a data availability statement. This statement should provide the following information, where applicable:

- Accession codes, unique identifiers, or web links for publicly available datasets
- A list of figures that have associated raw data
- A description of any restrictions on data availability

Raw and processed data files for human scRNA-seq data using both microfluidics-based and Seq-Well platforms are available for download through GEO under the accession number GSE137537 [https://www.ncbi.nlm.nih.gov/geo/query/acc.cgi?acc=GSE137537] and GSE137847 [https://www.ncbi.nlm.nih.gov/geo/query/acc.cgi?acc=GSE137847]. The count matrix, gene names, and sample annotation files analyzed in this study are also available as supplementary files.

# Field-specific reporting

Please select the one below that is the best fit for your research. If you are not sure, read the appropriate sections before making your selection.

☒ Life sciences ☐ Behavioural & social sciences ☐ Ecological, evolutionary & environmental sciences

For a reference copy of the document with all sections, see nature.com/documents/nr-reporting-summary-flat.pdf

# Life sciences study design

All studies must disclose on these points even when the disclosure is negative.

| | |
|---|---|
| Sample size | No predetermined sample sizes were performed. We analyzed retinal tissue from three individuals per group for both the microfluidics-based and Seq-Well platforms. This numbers of samples was sufficient to confidently annotate individual cell groups, and perform a data analysis. |
| Data exclusions | As described in methods, retinas collected for this study had no known retinal disease and no abnormalities indicative of disease pathology. A permissive quality control step was performed, followed by downstream analysis using the ACTIONet framework. Additional, putative low-quality cells were filtered out after post-processing as needed. More specifically, a manually curated set of marker genes encompassing all known major cell types in the retina was used to assess the putative identity of each cell. The value of each marker gene was imputed across all cells using a diffusion-based algorithm, and then individual markers corresponding to a given cell type were aggregated into a cell type-association score computed per each cell. A permutation test was subsequently used to assess the deviation from expectation of observed association scores. Cells that were either (i) not significantly associated with any cell type, or (ii) were significantly associated with more than one cell type, were filtered out from our study, due to the suspicion of potential doublets. Evidence of low quality of cells was additionally suggested by their isolated positioning in the network with respect to other highly clustering cells. For the rest of the cells, the cell type with the highest association score was selected as the most likely cell type annotation. Finally, the network context was used to filter out cells that were annotated with a given cell type, but their respective network neighbor cells were not statistically enriched with that same cell type annotation. We assume that such cells likely correspond to technical artifacts, such as doublets. For the macroglia cell subnetwork, cells marked as microglia were extracted and independently analyzed with the ACTIONet framework. Prior to analysis, mutual nearest neighbor was used again for batch correction to remove residual batch effects, and a subset of cells simultaneously expressing both macroglial and rod-specific markers potentially representing doublets was removed. |
| Replication | Single-cell RNA-seq findings were replicated across two platforms, microfluidics-based and Seq-Well. Verification of the single-cell RNA-seq data was performed for a subset of genes using fluorescent RNA in situ hybridization on human retinal tissue. |
| Randomization | Retinal tissue was not randomized. Covariates were controlled in the microfluidics single-cell RNA-seq analysis by obtaining postmortem eyes that are all male and isolated between 3.0-3.5 hour postmortem interval so that the samples are matched. Within this new scRNA-seq dataset, sex and postmortem interval are removed as drivers of variability. |
| Blinding | The investigators were blinded during quantification of the fluorescent in situ hybridization analysis. The single-cell RNA-sequencing analysis was not blinded. |

# Reporting for specific materials, systems and methods

We require information from authors about some types of materials, experimental systems and methods used in many studies. Here, indicate whether each material, system or method listed is relevant to your study. If you are not sure if a list item applies to your research, read the appropriate section before selecting a response.

## Materials & experimental systems

| n/a | Involved in the study |
|---|---|
| ☐ | ☒ Antibodies |
| ☒ | ☐ Eukaryotic cell lines |
| ☒ | ☐ Palaeontology |
| ☒ | ☐ Animals and other organisms |
| ☐ | ☒ Human research participants |
| ☒ | ☐ Clinical data |

## Methods

| n/a | Involved in the study |
|---|---|
| ☒ | ☐ ChIP-seq |
| ☒ | ☐ Flow cytometry |
| ☒ | ☐ MRI-based neuroimaging |

## Antibodies

| | |
|---|---|
| Antibodies used | Glutamine synthetase (BD Biosciences, 610518) was used to identify Muller glia. |
| Validation | The Glutamine synthetase antibody has been described and validated previously in publications, as well as by the company for IHC. We validated it in our work on human samples through co-localization with in situ hybridization probes specific for Muller glia. |

## Human research participants

Policy information about studies involving human research participants

| | |
|---|---|
| Population characteristics | We selected 10 individuals with no known ocular pathology; six for single-cell RNA sequencing and four for multiplex fluorescent in situ hybridization and immunofluorescence experiments. |
| Recruitment | No donors were recruited. Informed consent was obtained, and the postmortem eyes were obtained from autopsies at Massachusetts General Hospital (Boston, MA) as well as from the Alabama Eye Bank. |
| Ethics oversight | This study was approved by the Partners Institutional Review Board. |

Note that full information on the approval of the study protocol must also be provided in the manuscript.
