# Supplementary material for: Single-cell transcriptomic atlas of the human retina identifies cell types associated with age-related macular degeneration
Source: Nat Commun. 2019 Oct 25;10:4902. doi: 10.1038/s41467-019-12780-8 (PMC6814749; doi:10.1038/s41467-019-12780-8)
Supplement: Supplementary file 3 — Description of Additional Supplementary Files [file 41467_2019_12780_MOESM3_ESM.docx]

**Description of Additional Supplementary Files**

File Name: Supplementary Data 1.
Description: Quality control metrics of individual samples run using microfluidics and Seq-well scRNA-seq platforms.

File Name: Supplementary Data 2.
Description: Filtered Signature profile of 5,504 specific genes for the major cell types in the microfluidics-based platform. Rods, cones, retinal ganglion cells (RGCs), bipolar cells (BPs), amacrine cells (ACs), horizontal cells (HCs), macroglia, microglia, vascular cells.

File Name: Supplementary Data 3.
Description: Wilcoxon rank-sum test results for major cell types in the microfluidics-based platform.

File Name: Supplementary Data 4.
Description: Filtered Signature profile of 7,018 specific genes for the major cell types in the Seq-Well platform.

File Name: Supplementary Data 5.
Description: Wilcoxon rank-sum test results for the major cell types in the Seq-Well platform.

File Name: Supplementary Data 6.
Description: Signature profile of 6,305 specific genes for the macroglia subtypes in the microfluidics-platform.

File Name: Supplementary Data 7.
Description: Wilcoxon rank-sum test results for macroglia subtypes in the microfluidics-platform.

File Name: Supplementary Data 8.
Description: Wilcoxon rank-sum test for specificity of 585 AMD-associated genes in major cell types identified in the microfluidics platform.

File Name: Supplementary Data 9.
Description: Wilcoxon rank-sum test for specificity of 585 AMD-associated genes in major cell types identified in the Seq-Well platform.

File Name: Supplementary Data 10.
Description: Wilcoxon rank-sum test for specificity of 33 leading AMD-associated genes in the major cell types identified in the microfluidics platform.

File Name: Supplementary Data 11.
Description: Wilcoxon rank-sum test for specificity of 33 leading AMD-associated genes in the major cell types identified in the Seq-Well platform.
